# LIVING IN RURAL CONTEXTS: TOWARD A CRITICAL INTERDISCIPLINARY PERSPECTIVE ON RURAL AGING

**DOI:** 10.1093/geroni/igz038.1473

**Published:** 2019-11-08

**Authors:** Kieran Walsh, Mark Skinner

**Affiliations:** 1 NUI Galway, Galway, Ireland, Ireland; 2 Trent University, Peterborough, Ontario, Canada

## Abstract

Despite a growing focus on rural ageing, international literature in this field remains underdeveloped in critical and interdisciplinary perspectives. Reflecting traditional divisions across geographic, gerontological and health literatures, how we understand experiences of growing older in rural settings can still be characterised by a narrow, applied approach. This has implications for our capacity to disentangle multifaceted lived realities from rural contexts, and macro socio-economic and structural environments. There then remains questions about the ways in which the study of rural ageing needs to develop to direct policy, research and practice agendas to be a more critical reflection of these complexities. This symposium aims to draw together interdisciplinary critical perspectives on ageing and rurality as a means to advance this development. It will consider different theoretical approaches and major cross cutting challenges in relation to rural ageing. Burholt and Scharf will examine how critical gerontology has raised awareness of the heterogeneity of rural ageing across social justice elements of demography, resources, recognition and representation. Keogh and Walsh address these same elements in relation to the empirical intersection of exclusion and change in the production of a new rurality for older people. Cutchin and Rowles present a pragmatist theoretical perspective to encapsulate the essence of rural integration within an ever-changing milieu. Poulin et al. offer a critical approach to rural gerontological health that emphasizes intersectionality in the formation and development of older adult health. Herron and Skinner explore the intersectional construction of dementia and mental health in rural settings for older adults.

